# Operationalizing undifferentiated affect: Validity and utility in clinical samples

**DOI:** 10.3389/fpsyg.2022.690030

**Published:** 2022-11-10

**Authors:** Sean P. Lane, Timothy J. Trull

**Affiliations:** Department of Psychological Sciences, University of Missouri, Columbia, MO, United States

**Keywords:** ecological moment assessment, emotion differentiation, emotional granularity, generalizability theory, longitudinal data analysis

## Abstract

Emotion differentiation is conceptualized as the process of categorizing one’s general affective experiences into discrete emotions. The experience of undifferentiated affect or the inability to distinguish the particular emotion or combination of emotions that one is experiencing is often considered a hallmark of emotion dysregulation. Some past research has attempted to operationalize the general tendency to experience undifferentiated affect at the trait level using explicit questionnaire measures. More recently, indirect measures using intraclass correlation coefficients (ICCs) to estimate the consistency between simultaneous measures of different in-the-moment emotional experiences have become the favored method of quantifying undifferentiated affect. While the ICC method constitutes an advancement in estimating undifferentiated affect, which is theorized to be a dynamic process that occurs at a very granular level, prior investigations have used aggregate ICC measures or momentary ICC derivations that ignore multiple sources of dynamic variability to make inferences about in-the-moment experiences. We introduce a new, flexible method of calculating ICC measures of undifferentiated affect at different levels of experience that takes full advantage of time-intensive data measurement and more closely maps onto the theorized process. This method provides more refined estimates of undifferentiated affect and its associations with various behavioral outcomes, as well as uncovers more nuanced associations regarding the temporal process of emotional differentiation. It also elucidates potential conceptual issues in mapping empirical estimates of emotion undifferentiation onto their underlying theoretical interpretations.

## Introduction

Undifferentiated affect/emotion, alternatively characterized as the lack of emotional “granularity” or “complexity” (e.g., Suvak et al., 2011; Grühn et al., 2013; Kashdan et al., 2015), refers to an individual’s tendency to experience generalized feelings of positivity or negativity instead of actively discriminating between discrete emotional experiences (Barrett et al., 2001). This tendency is thought to inhibit one’s ability to regulate emotions and adapt to stress (Barrett et al., 2001) because the experience of discrete emotions provides information regarding appropriate coping behaviors (Schwarz and Clore, 1983, 2003). Indeed, the inability to differentiate between different affective experiences, especially negative ones (Barrett et al., 2001; Kashdan et al., 2015), has been linked to various behavioral and health impairments and clinical disorders (Kashdan et al., 2010; Demiralp et al., 2012; Pond et al., 2012; Selby et al., 2013; Zaki et al., 2013).

Recently, Kashdan et al. (2015) reviewed findings from research investigating processes linked to undifferentiated affect (UA), noting the large potential impact on general well-being. They also focused on the importance of careful measurement of UA, advocating experience-sampling or ecological momentary assessment (EMA; Stone and Shiffman, 1994; Shiffman et al., 2008) methodologies, which minimize retrieval and self-belief biases (Robinson and Clore, 2002) and allow for a covert behavioral index of UA to be calculated. Such an approach also allows for estimates of UA that are reflective of individuals’ actual day-to-day experiences. Moreover, others have noted that there has been a disconnect in the trait versus state theoretical conceptualization of emotion differentiation and its methodological operationalization, resulting in gaps in our empirical understanding of its effects on psychological outcomes (Thompson et al., 2021). However, depending on the conceptualization, matching the corresponding measurement allows for more robust tests of situational versus generalized hypotheses (Thompson et al., 2021). Recent meta-analyses speak to this gap, noting primarily small or nonsignificant findings between undifferentiation and well-being at the trait level and inconclusive patterns at the state level (O’Toole et al., 2020), or small but reliable negative associations between negative emotion differentiation and maladaptive behaviors specifically (Seah and Coifman, 2021).

We resonate with these reviews asserting that measurement of UA is important and that EMA is a promising way to characterize it (Kashdan et al., 2015), but that there is a limitation in pairing its conceptualization with its operationalization (Thompson et al., 2021) with emotional negativity being a particular area of interest (Seah and Coifman, 2021). The purpose of this paper is to demonstrate the limitations of commonly used analytic methods to quantify UA and how conclusions from previous ways of quantifying UA may, in fact, be misleading. Specifically, we highlight how previously used analytic methods, essentially person level analytic approaches, can result in the inflation of UA estimates because they do not separate or identify variance due to systematic changes in affect, variation in difficulty of items, and variance due to items measuring the same subscale of affect. To address these limitations, we offer an analytic method using Generalizability Theory (GT; Cronbach et al., 1972; Brennan, 1992; Cranford et al., 2006) to partition these important sources of variance. If multiple items per emotion category are administered, this method can be used to estimate UA at the *momentary level* in intensive longitudinal data, a level where UA processes are theorized to operate (Barrett et al., 2001; Erbas et al., 2019). We discuss the decision-making process regarding how to handle assessment data that include the identical ratings for items (resulting in a lack of variance), a result that is more likely when considering momentary data. Finally, we compare how the associations between UA and self-reported impulsive behaviors differ when operationalizing UA, (a) with traditional versus GT approaches, and (b) at different levels of experience (i.e., momentary-, daily-, and person-level). We also consider the implications of handling assessments with no variability in items’ scores in different ways.

### Generalizability theory

Generalizability Theory (GT) offers a way to systematically examine the variance in single-trial occasion measurements and identify various sources of systematic signal that can be used to estimate reliability coefficients. GT is an expansion of classical test theory (i.e., Spearman-Brown approaches), which acknowledges that the variance in an observed score is born of multiple influences (Cronbach et al., 1972). GT decomposes a true score into a researcher-specified set of constituent systematic sources of variance based on the structure of their data rather than a single true score as in classical test. In this way, GT can be used to better capture and understand not only factors conceptualized as contributors to randomness, but also how variance components of interest may affect observed scores (Shrout and Lane, 2012). Within the GT framework, the first set of analyses conducted is commonly referred to as a “G study,” the goal of which is to estimate sources of potential variance in observed scores. An advantage of this approach is that the variance components included in the G study can be defined and adjusted by the researcher according to their research design and desired application. Further, the treatment of repeated measures within subjects can be specified (e.g., crossed vs. nested within subject) when estimating these variance components. G study analyses are computed as linear combinations of ANOVA mean squares.

The second set of analyses utilized in GT is commonly referred to as the “D study,” wherein the variance components derived from the G Study are used to estimate reliability as a proportion of variance. Just as in classical test theory, the reliability (i.e., dependability and consistency) of a measurement represents the ratio of variance from “true” scores to the total relevant variance (i.e., true variance + variance attributable to all sources of error). Factors are treated as either fixed or random by their inclusion in the denominator of the reliability formula. These analyses are used to assess the reliability of a measurement given a study’s assessment factors (e.g., survey assessments varied across raters, items, days, etc.). It is here where the ICCs that represent undifferentiated negative affect are calculated based on the set of derived variance components.

### Person level UA estimation

Most investigations of UA involving intensive longitudinal assessments have relied on indirect average inter-item correlations (e.g., Zaki et al., 2013; Erbas et al., 2018; Kalokerinos et al., 2019) or intraclass correlation coefficients (ICCs; e.g., Pond et al., 2012) to evaluate the degree to which responses to items corresponding to different emotions from self-report questionnaires are consistently rated similarly in generating an index of UA. ICCs subsume inter-item correlations, so we focus on those as our point of comparison. The common design is to ask individuals to rate, using self-report, and the same series of affect items across multiple occasions or days. Then a basic two-way ANOVA model corresponding to Equation 1 can be estimated for each person where items are treated as fixed and occasions as random.


(1) *Ratingi_o_ = μ + Ii + O_o_ + ei_o_*

Here, *Rating_io_* corresponds to a person’s rating of item, *i*, at occasion, *o*. μ is the grand mean for all ratings. *I_i_* is the tendency for an item to be rated higher/lower on average for that person, and *O_o_* is the tendency for a given occasion to on average have higher/lower ratings. *e_io_* is error. Once this model is estimated, a traditional ICC can be computed using the sums of squares [specifically ICC(3,1); Shrout and Fleiss, 1979]. The resulting value is an index of the consistency with which an individual rates items across occasions. Higher values indicate that an individual does not vary in the way he/she rates items at a given random occasion (i.e., high UA).

This approach seems straightforward. However, we note a few important limitations. First, if individuals are being sampled over multiple days or weeks where systematic effects on affect might be expected (i.e., weekend, morning/evening), *estimates of UA may be inflated due to the confounding of occasion and error*. Second, if certain items are systematically elevated across occasions as a result of being easier to endorse (e.g., sad vs. hopeless), *UA estimates may again be inflated because this variance is being confounded with variance estimated across items* (i.e., precisely what we are most interested in as an indicator of UA). Third, there may be *other systematic factors that contribute variance to the error*, independent of individual items and occasions, which may inflate estimates (e.g., if certain items correlate because they belong to a common subscale). Each of these factors can effect overall UA estimates as well as associations between UA and other variables to the extent to which individuals vary on their experience of each (i.e., some individuals experience more extreme diurnal and/or weekly shifts in mood than others).

To account for these potential confounding factors, we adopt a GT approach (Cronbach et al., 1972; Brennan, 1992; Cranford et al., 2006) to estimating ICCs for UA. The GT approach uses the same ANOVA structure to model variability, except it allows the basic two-way ANOVA model to be expanded to accommodate other sources of variance. Such models can then be estimated using any variance decomposition software (for examples, see Shrout and Lane, 2012) and the individual variance components used to estimate ICCs. We start by giving the analogous GT ICC estimate for UA based on the ANOVA model in Equation 1. It is given in Equation 2.


(2) $$R_{P1}=\frac{\sigma_{ITEM}^{2}}{\left(\sigma_{ITEM}^{2}+\sigma_{ERROR}^{2}\right)}$$

This estimate gives the proportion of total variance that is accounted for by individual items (i.e., how differentiated each item is from the others across repeated measurements) and is analogous to ICC(3,1). To get an estimate of UA, we subtract this value from one. Next, we expand the ANOVA model to include other sources of variance. We do this based on an EMA design (see below) in which individuals are assessed multiple times a day for a number of consecutive days. At each assessment, they complete a set of items (e.g., negative affect) that contain item subsets corresponding to different specific emotions (e.g., sad, hostile, and fearful). Using this design, the following ANOVA model can be constructed (Equation 3).


(3) *Rating_io_ = μ + I_i_ + S_s_ + O_o_ + D_d_ + (IS)_is_ + (IO)_io_ + (ID)_id_ + (SO)_so_ + (SD)_sd_ + (OD)_od_ + (ISO)_iso_ + (ISD)_isd_ + (IOD)_iod_ + (SOD)_sod_ + e_isod_*

The interpretation is as before, except now we have the additional factors of subscale, *s*, and day, *d*. There are also the six two-way and four three-way interactions between the variables. This allows for certain subscales and certain days to have systematically higher/lower ratings than others across the repeated assessments. The two-and three-way interactions allow, for example, that certain subscales may be systematically higher/lower on specific days (e.g., if hostility is particularly low on weekends compared to sadness and fear).^1^

After performing variance decompositions for each individual and saving the variance components, an estimate for UA can be calculated using Equation 4, where *N* is the number of items per subscale.^2^


(4) $$R_{P2}=\frac{\sigma_{SUBSCALE}^{2}+\sigma_{ITEM*SUBSCALE}^{2}}{\left(\sigma_{SUBSCALE}^{2}+\sigma_{ITEM*SUBSCALE}^{2}+\frac{\sigma_{ERROR}^{2}}{N}\right)}$$

This estimate gives the proportion of total variance that is accounted for by the different subscales (i.e., how consistently an individual rates items within a subscale as compared to items that belong to different subscales). This value represents a generalized estimate of ICC(3,1) generated as the *D*-study portion of a generalizability analysis (Cronbach et al., 1972). We again subtract this value from one to get an estimate of UA. If an investigator is treating items as nested, the variance for the interaction terms in the equation would be dropped and the item nested with subscale variance (*I_i(s)_*) may be added to the denominator.

### Momentary UA estimation

It seems desirable to examine UA as it occurs in daily life, and how it, itself, may be dynamic. However, previous operationalizations of UA calculate person-level estimates to characterize this theoretically very temporally fleeting process (Barrett et al., 2001; Zaki et al., 2013; Erbas et al., 2018; Kalokerinos et al., 2019). Momentary UA can be evaluated when multiple items are used to assess each of a set of emotions in a given assessment. Then a model analogous to Equation 1 can be estimated, but instead this is done for each assessment in an individual’s time series.


(5) *Rating_is_ = μ + Ii + Ss + e_is_*

Equation 5 depicts the model fit for each occasion of an individual. At each time point, a rating is a function of the grand mean (μ), which item is being responded to (*i*), and which subscale that item belongs to (*s*). The corresponding estimate for the momentary ICC is as follows:


(6) $$R_{M1}=\frac{\sigma_{SUBSCALE}^{2}}{\left(\sigma_{SUBSCALE}^{2}+\frac{\sigma_{ERROR}^{2}}{N}\right)}$$

The formula for *R_M1_* is similar in structure to *R_P1_* except the item variance has been replaced with subscale variance as in *R_P2_*. In addition, we divide the error by the number (*N*) of subscale items as in *R_P2_*, since our interest is in differences in ratings across subscales. Again note that if items are considered nested within subscale, item variation would be included in the denominator. As in *R_P2_,* we must again subtract this value from 1 to get an estimate of UA.

Of note, and core to the labeling of the index as undifferentiated *affect*, the resulting ICC represents individuals’ tendencies to disambiguate general affective categories and not the specific emotions that may comprise those categories, such as those located within the same quadrants or cluster regions of the affective circumplex (Russell, 1980). The theory of *emotion* differentiation (Barrett et al., 2001) argues for individuals’ ability or tendency to isolate discrete emotional experiences, as it serves to inform emotion regulation strategies. This is how all operationalizations of emotion differentiation have proceeded to date, either at the person (i.e., trait) or occasion level. However, we consider at least two related challenges to the validity of these approaches. First is that research suggests that, at least when utilizing self-report in naturalistic environments, individuals tend not to discriminate reliably between individual emotion prompts, but rather very reliably group them into broader affective categories (e.g., Hepp et al., 2017, 2018, 2020; Erbas et al., 2019). Second, existing approaches use individual emotion items to estimate emotion differentiation indices that assume perfect reliability (i.e., no measurement error), yet psychometrically and empirically single item measures underperform (e.g., Wanous and Reichers, 1996; Wanous and Hudy, 2001). Combined, these methodological concerns would suggest that existing emotion differentiation estimation approaches are contaminated by and possibly capitalizing on considerable error, drawing into question the various empirical associations that have been observed, or at least if they represent true differentiation or some degree of response bias. By utilizing multiple items to assess broader affective categories and broadening the scope of differentiation to the disambiguation of those categories, the current proposed method addresses these issues and increases the reliability of the (un) differentiation estimate and resulting inferences. However, then the construct being measured conceptually changes.

One potential limitation of estimating UA at the momentary level is if there is no variability in ratings (e.g., if all items are rated as 0, or otherwise absent, in the moment). If all ratings are the same, at least superficially that would appear as complete undifferentiation, since an individual is not discriminating between different discrete emotion probes. This seems reasonable if all of the ratings are elevated indicating at least some emotional experience. However, it is less clear in the case that all of the ratings are at the floor of a given scale (i.e., a score of 0, or otherwise “not at all” on many scales), usually indicating the absence of experiencing the given emotion. We examine the frequency of such reports and their impact on analysis results and interpretations in the following example. At any rate, empirically, using our momentary method would result in missingness in each of these scenarios and decisions must be made on how to handle it. We discuss three options based on the above rationale.

## Empirical example

The current example uses an EMA approach to examine the relationship between undifferentiated negative affect (UNA) and impulsivity/substance use among individuals with disorders often tied to emotion dysregulation. Past studies have found that UNA is often associated with general predispositions toward impulsivity (Tomko et al., 2015), as well as specific impulsive behaviors (Kashdan et al., 2010; Pond et al., 2012; Selby et al., 2013; Zaki et al., 2013). We estimated UNA at both the person level and momentary level using Equations 2, 4, 6, including three ways of handling missing data at the momentary level due to the absence of variability. Results from analyses predicting impulsivity and substance use are compared across the different methods. A simulation study was also conducted to corroborate empirical findings.

## Materials and methods

### Participants

Participants^3^ included 131 individuals with borderline personality (*N* = 81) and depressive (*N* = 50) disorders who were recruited from local psychiatric outpatient clinics for a study examining affective instability (see Trull et al., 2008). Previous studies have reported on differences between these two diagnostic groups in terms of mean levels (e.g., Trull et al., 2008; Solhan et al., 2009; Tomko et al., 2014, 2015) and associations between (Jahng et al., 2011) variables that we include in our analyses, including UNA (Tomko et al., 2015). While there were mean level differences in some of these variables between groups, specifically UNA (Tomko et al., 2015), there were no differences in the associations between these variables across diagnostic groups in the presented analyses, so we chose to combine the data across groups. This quality, with respect to the association between emotion (un) differentiation salient outcomes speaks to its theorized transdiagnosticity as a construct across healthy and dysregulated emotional functioning (Barrett et al., 2001).

**Figure 1:**
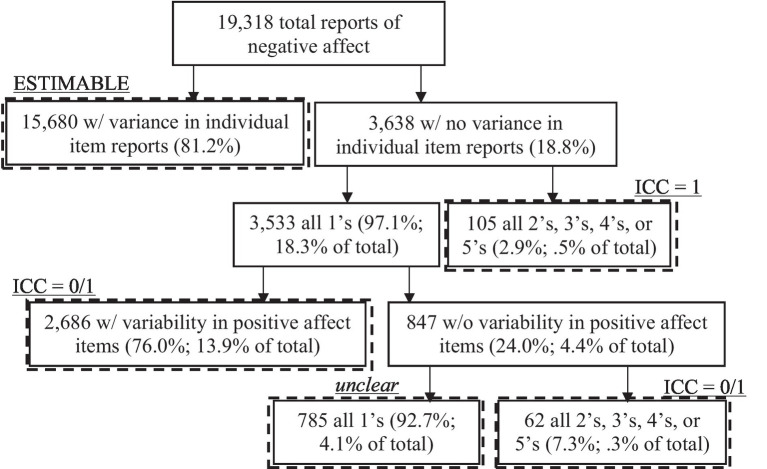
Flow diagram of affect reports with and without variability in item ratings. Undifferentiated affect cannot be estimated at the occasion level for affect reports with no variability. Therefore decisions must be made to exclude such reports from analysis, or impute them with theoretically meaningful values. We present potential options, though it is unclear how to handle occasions with no variability across all items of both positive and negative affect.

Given these two groups’ chronic elevated experience of negative affect compared to the general population, we viewed analysis of their data as an example of a *minimal-impact* case of the influence of zero variance reports. Zero variance reports, for example, may result from ratings indicating no experienced negative affect (i.e., all floor reports; see Figure 1). Analysis of UNA in the general population compared to our sample would likely result in more zero-variability reports and less variability in ratings overall.

General exclusion criteria included having a psychotic disorder, history of severe head trauma, intellectual disability, severe substance dependence, or severe neurological dysfunction. Individuals were required to be between the ages of 18 and 65 to participate (*M* = 31.6, *SD* = 11.9). Most participants were female (92.5%), of Caucasian ethnicity (82.1%), were single/never married (53.7%), had an annual income less than $25,000 (74.6%), and had current comorbid anxiety (85.9%) or mood (63.1%) disorders.

### Procedure

Participants who passed an initial eligibility screening were scheduled for an orientation session where diagnostic information was obtained from semi-structured interviews (Pfohl et al., 1994; First et al., 1995; see Trull et al., 2008 and Tomko et al., 2015, for details). After being confirmed as eligible, participants were issued an electronic diary (Palm Zire 31© handheld computer) that they carried for approximately 28 days (*M* = 28.8 days). The electronic diary (ED) alarmed six times per day, prompting the individual to answer questions about current mood and a variety of different substance use. Across prompts, the item sets corresponding to different modules were always administered in the same order, as were items within each module. However, items for modules that represented scales were *a priori* randomized (e.g., intermixing positive and negative affect items and affect items within individual subscales). The alarm times were determined by a software program that stratified the participants’ usual waking hours (as reported by the participant prior to the study start) into six equal intervals, and then randomly selecting a time within each interval (see Trull et al., 2008, for more details regarding the electronic diary protocol). The compliance in the sample was high (*M* = 85.8%), with participants completing an average of 147.0 prompts each. In total, 19,318 prompts were completed and included in the analyses.

### Measures

#### Negative affect

Affect was assessed using items from the Positive and Negative Affect Schedule-Extended version (PANAS-X; Watson and Clark, 1999). Items were presented to each participant on the ED during each of the six daily momentary assessments. For each affect item, respondents were asked to rate the extent to which they felt the particular affective state on a five-point Likert scale (1 = very slightly or not at all to 5 = extremely) since the last prompt. The negative affect items composed three negative emotion scales: fear (six items; afraid, nervous, frightened, shaky scared, and jittery), hostility (six items; angry, irritable, hostile, loathing, scornful, and disgusted), and sadness (five items; sad, blue, alone, downhearted, and lonely). Overall level of negative affect was created as an average of the 17 items. It is important to note that both the average individual differences in these subscales (*R_KF_*’s > 0.95) and their reliabilities of change across time points (*R_C_*’s > 0.84) have high reliability (Shrout and Lane, 2012; Hepp et al., 2017), given that the current ICC approach explicitly parses variance assumed to be unique across items that belong to different subscales. In the case where individual item or subscale ratings were not reliable, (un) differentiation estimates would similarly not be.

#### Undifferentiated negative affect

To estimate an individual’s negative affect undifferentiation, variance decomposition analyses were conducted at either the person-or occasion-level in accordance with Equations 2, 4, 6. At the *person level*, models were specified according to Equation 2 for the conventional UNA ICC, and Equation 4 for the GT-based UNA ICC. At the *occasion level*, a model based on Equation 6 was specified. The variance components from each model were then used to estimate person-level UNA ICCs for the conventional (Equation 2) and GT (Equation 4) approaches, as well as individual ICCs for each occasion for each individual (Equation 6). Figure 1 shows a flow diagram of occasions in which momentary ICCs were (in) estimable and potential options for imputation. In general, we note that in our sample only 4.1% of prompts were characterized by no variability across all negative and positive affect items. This suggests that the 13.9% of prompts that had no variability in negative affect ratings but some variability in positive affect ratings were likely veridical (negative and positive items were intermixed). Were instances of no variability assumed to represent complete undifferentiation, as in conventional person-level (Barrett et al., 2001) as well as newer momentary (Erbas et al., 2022) approaches, no data would be lost in analyses. We note that this is based on what is assumed by the calculation of the index, not the theoretical conceptualization of UNA. Similarly, if assumptions were made regarding what those observations theoretically represented, no data would be lost. But if researchers were unwilling to make such assumptions, up to 18% (*n* = 3,477) of data points would be missing and is an open area of methodological consideration (Erbas et al., 2022). We implement each of these three scenarios to examine the impact on results.

We additionally estimated undifferentiated negative *emotion* using the approach proposed by Erbas et al. (2022) as a momentary comparison index. However, in using this approach, we re-estimated disattenuated (Spearman, 1904, 1910) reliability indices for single items based on the 17 items used in our analyses, and the corresponding values indicated excellent reliability for person-level estimates (*R_KF_* = 0.99) but substantially lower, marginal reliability for momentary estimates (*R_C_* = 0.52). Moreover, we note that this approach confounds item-specific and subscale-shared variance and so where the undifferentiation signal is originating, and in what proportions, is unknown (c.f., Erbas et al., 2019). As a result, we expected potentially small associations with our momentary index, as well as small associations with conventional indices, as previously reported.

#### Momentary impulsivity

At each prompt, participants were asked to rate their impulsivity since the last prompt. Participants responded to four items using a five-point Likert scale (1 = very slightly or not at all; 2 = a little; 3 = moderately; 4 = quite a bit; and 5 = extremely; Momentary Impulsivity Scale; MIS; Tomko et al., 2014, 2015). The individual items were, “I made a ‘spur of the moment’ decision,” “I said things without thinking,” “I spent more money than I meant to,” and “I have felt impatient.” Items were summed to create a total score, which was reliable both at the between-person (*R_KF_* = 0.98) and within-person (*R_C_* = 0.81) level (Shrout and Lane, 2012).

#### Substance use

At each prompt, participants indicated if they had used caffeine, tobacco, alcohol, or marijuana since the last prompt (1 = yes, 0 = no). In total 128 (97.7%) individuals reported using caffeine at least once during the diary period, 75 (57.3%) reported using tobacco, 90 (68.7%) reported using alcohol, and 35 (26.7%) reported using marijuana. In total, there were 5,788 reports of caffeine use, 6,919 reports of tobacco use, 948 reports of alcohol use, and 821 reports of marijuana use.^4^ Results did not differ whether we limited the analyses for each substance just to users of the specific substance or included the entire sample. We report results for analyses using the entire sample to facilitate the comparability of results across substances.

### Data analysis

In all analyses, UNA was indexed using the various ICCs described above. Five sets of person level linear regressions were first conducted using aggregate measures of the dependent variables, an aggregate measure of negative affect, and either aggregated occasion level estimates of UNA or person level estimates of UNA.

Aggregates were estimated as the average across all prompts for a given individual. As a result, all substance aggregates represented the percentage of prompts that an individual reported using each individual substance. Impulsivity and negative affect were representative of the average level of each variable, reported across the entire EMA period. As denoted in Equations 2, 4, the conventional and full GT ICC estimates, respectively, were explicitly estimated at the person level, so no aggregation was necessary. The occasion level ICC estimates (Equation 6) were aggregated similar to impulsivity and negative affect. All covariates were centered on the sample means.

Next, three sets of momentary analyses were conducted using the momentary indices of UNA with different treatments of missing values (undifferentiation imputed, left missing, and conditionally imputed). When there was no variance across item ratings for a given occasion, we either (a) imputed as complete undifferentiation (i.e., a value of 1), (b) set it as missing, or (c) conditionally imputed responses as complete undifferentiation if all of the ratings were elevated but there was no variance (i.e., all negative affect items were rated as either 2, 3, 4, or 5) *but* as complete differentiation (i.e., a value of 0) if all of the item ratings were 1 (i.e., “not at all”). Given that momentary affect data were collected at multiple occasions within days and across persons, there are three levels at which UNA could be measured (c.f. Curran and Bauer, 2011). Correspondingly, we calculated ICC measures of UNA for each individual, (1) at each occasion, (2) on each day, as the average of the occasion-level ICCs for that day, and (3) as a person average across the daily averages of the diary period. We calculated similar scores for negative affect. For impulsivity we then fit a multilevel model corresponding to:


(7) MIS_ijk_ = (b_0_ + b_0i_) + b_1_*ICC_occasion_ijk_ + (b_2_ + b_2i_)*ICC_day_ij_ + b_3_*ICC_personi + (b_4_ + b_4i_)*NA_occasion_ijk_ + (b_5_ + b_51_)*NA_day_ij_ + b_6_*NA_personi + e_ijk_

In this equation, *MIS_ijk_* is the momentary impulsiveness rating of person *i* on day *j* at occasion *k*. There is a global intercept (*b_0_*) as well as a person-level intercept (*b_0i_*) such that across the diary period some individuals might report more impulsivity than others on average. Next there is an effect of undifferentiated negative affect at the occasion-level (*b_1_*) on occasion-level impulsivity. This effect describes the degree to which feeling undifferentiated negative affect in the moment relates to concurrent reports of impulsivity in the moment. Similarly, there is a between-person effect of an individual’s average ICC for a given day (*b_2_*) and the corresponding person-specific random effect (*b_2i_*). These effects describe the degree to which feeling undifferentiated negative affect on average on a particular day is related to higher reports of impulsivity at some point throughout that day. Then, there is the effect of an individual’s average 28-day ICC on impulsivity at a given occasion (*b_3_*). This represents the extent to which someone who is on average undifferentiated with respect to their reports of negative emotions also reports more impulsivity at any given occasion. This is analogous to a trait-level or personality effect.

Analogous to the ICC effects, there are corresponding effects for occasion-level negative affect (*b_4_* and *b_4i_*), day-level negative affect (*b_5_* and *b_5i_*), and person-level negative affect (*b_5_*). Lastly, there is an error term (*e_ijk_*). Each of the covariates were centered such that occasion-level variables were centered on the person-average for that day, day-level variables were centered on the person-average of day-averages for that person across the diary period, and person-level variables were centered on the average of person-averages across the diary period (see Tomko et al., 2015, for full descriptions of parameters).

At the momentary level, since the substance use variables were binary, we opted to fit logistic models using Generalized Estimating Equations (GEE; Liang and Zeger, 1986), in which there are no random effects estimated, but instead clustering is adjusted for in the residual covariance matrix through estimation of robust standard errors (see Burton et al., 1998; Carlin et al., 2001; Hubbard et al., 2010 for discussions comparing GEE and multilevel approaches with continuous versus categorical outcomes).^5^ Data preparation and analysis syntax, including example data, for the presented results are available *via* https://osf.io/ftwrs/.

## Results

The BPD group had higher undifferentiated negative affect estimates using the conventional ANOVA [*M_BPD_* = 0.74, *M_DD_ = 0*.65, *t*(129) = 2.60, *p* = 0.010], conventional GT [*M_BPD_* = 0.80, *M_DD_ = 0*.71, *t*(129) = 2.86, *p* = 0.005], full GT [*M_BPD_* = 0.59, *M_DD_ = 0*.48, *t*(129) = 2.27, *p* = 0.025], undifferentiation imputed [*M_BPD_* = 0.71, *M_DD_ = 0*.64, *t*(129) = 2.12, *p* = 0.036], and imputed missing [*M_BPD_* = 0.64, *M_DD_ = 0*.59, *t*(129) = 1.79, *p* = 0.076] estimates, but not for the conditionally imputed [*M_BPD_* = 0.51, *M_DD_ = 0*.48, *t*(129) = 0.76, *p* = 0.451] estimates. Consistent with these results, apart from the conditional imputation, the BPD group had higher undifferentiated negative affect estimates using the Erbas et al. (2022) method [*M_BPD_* = 6.07, *M_DD_ =* 5.20, *t*(129) = 1.98, *p* = 0.050].

BPD individuals were also more likely to be marijuana users [χ^2^(1) = 4.74, *p* = 0.029] and slightly more likely to be tobacco users [χ^2^(1) = 2.83, *p* = 0.093]. Despite these average level differences in the independent and dependent variables between the two groups, we did not observe group differences in the associations between them, and so we collapsed all reported results across the two groups for ease of presentation.

### Comparing empirical undifferentiated negative affect estimates for (1) conventional person level, (2) full GT person level, and (3) person aggregated occasion level approaches

In the top portion of Table 1, we first report the person-level estimates of UNA using the conventional ANOVA-based approach as reported in other studies (Tugade et al., 2004; Kashdan et al., 2010; Pond et al., 2012; Grühn et al., 2013), followed by the analogous estimates using GT (with only item and measurement occasion as factors in the model) to demonstrate the comparability of the models, and lastly, include the estimates of the full GT model, which estimates variances for all levels of measurement, including their interactions. The conventional ANOVA and GT estimates are not different, demonstrating that the two approaches are analogous. The UNA estimates from the full GT model are substantially smaller, indicating more differentiation across individuals on average, but still do correlate quite highly with the conventional estimate (*r* = 0.81).

**Table 1:** Person-level descriptive statistics and bivariate correlations for different operationalizations of undifferentiated affect.

		ICC	*M*	*SD*	1	2	3	4	5	6	7
Person level	1.	Conventional – ANOVA	0.71	0.19	1.00						
	2.	Conventional – GT	0.71	0.19	**1.00**	1.00					
	3.	Full GT	0.55	0.28	**0.81**	**0.81**	1.00				
Occasion level aggregated to person level	4.	Erbas et al. (2022)	5.74	2.47	0.04	0.04	−0.02	1.00			
	5.	Zero var. imputed w/ 1	0.68	0.19	**0.73**	**0.73**	**0.81**	−0.13	1.00		
	6.	Zero var. set to Missing	0.62	0.18	**0.56**	**0.56**	**0.76**	−0.16	**0.92**	1.00	
	7.	Zero var. imputed w/ 0 for all 1’s, 1 for all 2’s-5’s	0.50	0.18	0.03	0.03	**0.31**	−0.06	**0.38**	**0.68**	1.00

ICC, intraclass correlation; GT, Generalizability Theory; and Var, variance. ICC values of 1 index complete undifferentiation while values of 0 index complete differentiation. Bold values are significant at *p* < 0.001.

In the bottom portion of Table 1, we present the occasion-level undifferentiated negative affect estimates that are then aggregated to the person-level, including the method of Erbas et al. (2022) proposed for estimating undifferentiation as a comparison. However, due to the issue of inestimability when there is no variance across item ratings for a given occasion, we present the average values and correlations when those occasions were, (a) imputed as complete undifferentiation (i.e., a value of 1; *N* = 3,638, Figure 1) in line with what the person-level models implicitly assume and consistent with previous conceptualizations, (b) set to missing and effectively ignored, reducing overall sample size (Tomko et al., 2015), and (c) conditionally imputed as complete undifferentiation if all of the ratings were elevated but there was no variance (i.e., all negative affect items were rated as either 2, 3, 4, or 5; *N* = 105, Figure 1) *but* as complete differentiation (i.e., a value of 0; *N* = 3,533) if all of the item ratings were 1.^6^

When occasions with zero variance are treated as completely undifferentiated they correlate most highly with the full GT person-level estimates (*r* = 0.81) and somewhat less, but still strongly with the conventional approach (*r* = 0.73). When occasions with zero variance are excluded from the aggregated occasion-level estimates (18.8% of all occasions, Figure 1), as expected, those correlations are reduced (*r* = 0.56). However, when zero-variance occasions are conditionally imputed (i.e., 97.1% of which are coded are complete differentiation) the correlations with the undifferentiation estimates from the conventional approach are near zero (*r* = 0.03), and the correlations with the full GT estimates and conceptually similar undifferentiation imputed occasion-level aggregates are small to medium (*r*s = 0.31 and.38, respectively). In general, we see that both person-level and our aggregated momentary GT estimates are generally unrelated (and possibly negatively) to the aggregated person-level estimates using the Erbas et al. (2022) approach.

The reasons for the differences between these approaches are 2-fold. The first is a result of systematic variance in item ratings that is due to other sources that are either ignored, as in the case of the conventional approach (e.g., Scale^*^Item, *Day*), or confounded with error due to the level of analysis, as in the occasion-level approach (e.g., Subscale^*^Occasion^*^Day). The second is due to the theoretical meaning and subsequent treatment of measurement occasions that have no variance. As shown by Figure 1, those instances, instead of communicating UNA in the cases when negative affect is in fact elevated, could be indicative of a quite differentiated *lack* of negative affect. We return to this second consideration in the discussion, as it is grounded more in theory than what is empirically estimable, and may have implications for the interpretation of previously reported results. Next, we seek to quantify the impact of not taking into account systematic factors that might affect the scale and error variance components that will then lead to bias in the UNA ICC estimates.

### Simulation demonstrating the impact of additional sources of variance on undifferentiated affect estimates

We can see from Table 2 that using individual items as the level of measurement in the conventional approach leads to an overestimation of the amount of variance that is due to differences in particular negative affect subscales. This is primarily due to certain items within each subscale being systematically rated higher/lower across all ratings (σ^2^_Scale*Item_ = 0.139). Similarly, using overall measurement occasion in the conventional approach ignores that there might be systematic variance due to specific days (e.g., weekends), occasions (e.g., mornings), or particular occasions given the day (e.g., Sunday night before work Monday morning). We find that day (σ^2^_Day_ = 0.100; 11.1%) and occasion-by-day (σ^2^_Occ*Day_ = 0.092; 10.2%) account for approximately equal amounts of variance. Lastly, there are multiple systematic sources of variance that could otherwise inflate the amount of error that is estimated using the conventional approach. The largest of those estimated using our example data was that due to certain subscales being rated higher/lower overall, on specific occasions, on certain days (σ^2^_Scale*Occ*Day_ = 0.071; 7.9%). Importantly, the presence of any of these sources of variance within a given participant’s data could bias the resulting estimate of person-level differentiation in different ways.

**Table 2:** Person-level variance decompositions of emotion ratings.

Variable	Conventional		Full GT	
	*M* (*SD*)	%	*M* (*SD*)	%
σ^2^_Item_	0.231 (0.299)	27.5%	0.020 (0.040)	2.2%
σ^2^_Scale_	–		0.115 (0.258)	12.8%
σ^2^_Scale*Item_	–		0.139 (0.192)	15.4%
σ^2^_Measurement_	0.225 (0.271)	26.9%		
σ^2^_Occ_	–		0.004 (0.010)	0.5%
σ^2^_Day_	–		0.100 (0.178)	11.1%
σ^2^_Occ*Day_	–		0.092 (0.117)	10.2%
σ^2^_Error_	0.382 (0.269)	45.6%	0.237 (0.188)	26.4%
σ^2^_Scale*Occ_	–		0.003 (0.006)	0.2%
σ^2^_Item*Occ_	–		0.001 (0.002)	0.1%
σ^2^_Scale*Day_	–		0.042 (0.057)	4.7%
σ^2^_Item*Day_	–		0.005 (0.010)	0.6%
σ^2^_Scale*Item*Occ_	–		0.005 (0.008)	0.5%
σ^2^_Scale*Item*Day_	–		0.058 (0.065)	6.5%
σ^2^_Scale*Occ*Day_	–		0.071 (0.061)	7.9%
σ^2^_Occ*Item*Day_	–		0.007 (0.012)	0.8%
σ^2^_Total_	0.838 (0.660)	100.0%	0.899 (0.724)	100.0%
ICC	0.768 (0.174)		0.550 (0.277)	

Estimated variances will not add up exactly because they are averages of person-level estimates. However, the item variation using the conventional approach (σ^2^_Item_ = 0.231) was similar to the sum of the item, type, and type-by-item variation using the GT-based approach, which mathematically it subsumes (σ^2^_Item_ + σ^2^_Type_ + σ^2^_Type*Item_ = 0.020 + 0.115 + 0.139 = 0.254), as were the comparable measurement (σ^2^_Conventional_ = 0.225; σ^2^_GT_ = 0.196), error (σ^2^_Conventional_ = 0.382; σ^2^_GT_ = 0.429), and total variances (σ^2^_Conventional_ = 0.838; σ^2^_GT_ = 0.899). Using the median variances also provided similar estimates (item—σ^2^_Conventional_ = 0.113; σ^2^_GT_ = 0.092; measurement—σ^2^_Conventional_ = 0.133; σ^2^_GT_ = 0.071; error—σ^2^_Conventional_ = 0.328; σ^2^_GT_ = 0.314; total—σ^2^_Conventional_ = 0.698; σ^2^_GT_ = 0.746). Furthermore, the total variances for each method were nearly perfectly correlated (*r* = 0.996), demonstrating that although the methods obtained slightly different estimates due to the different assumed models, they provided consistent estimation across persons.

**Figure 2:**
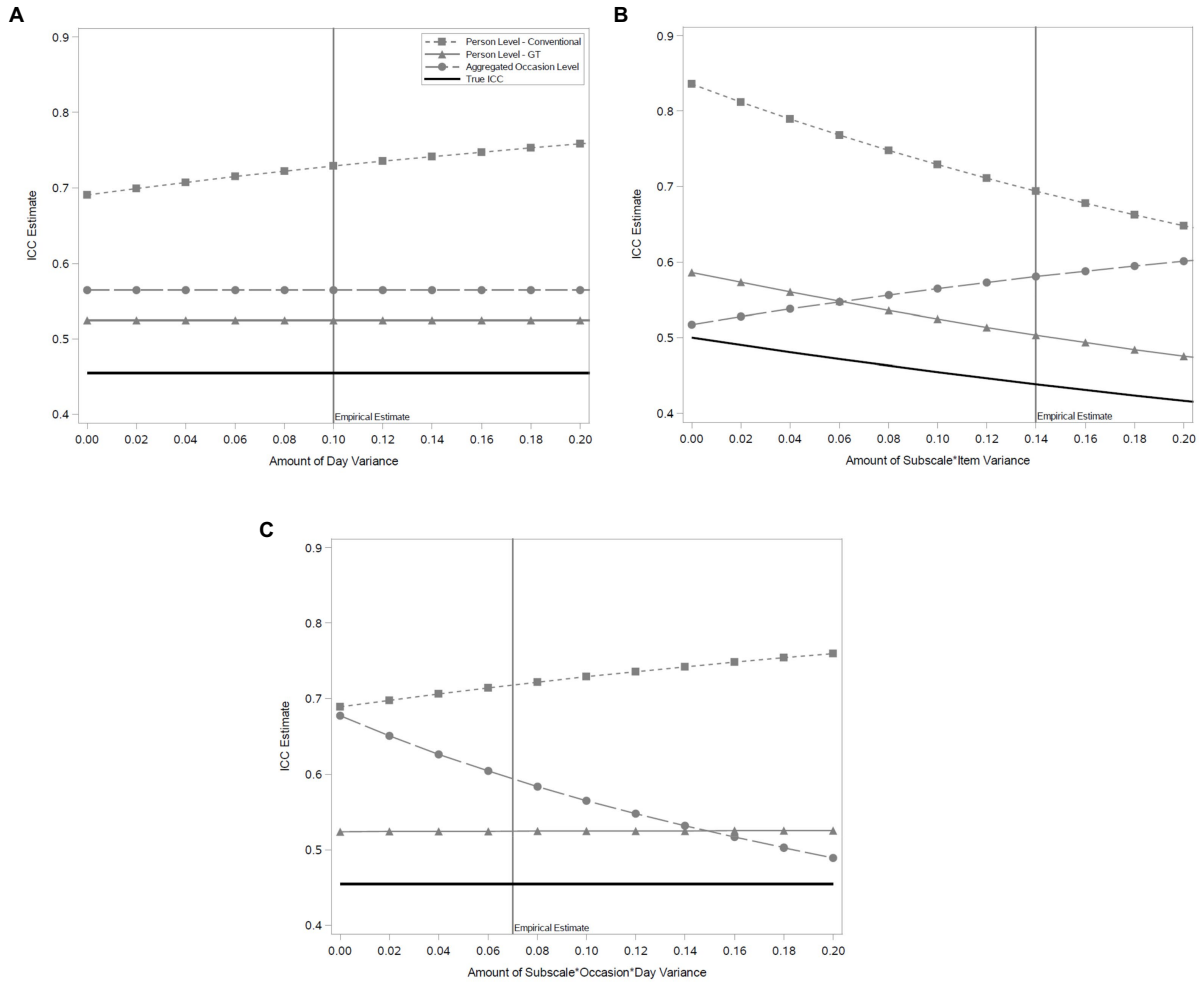
Simulation results for conventional, Generalizability Theory, and aggregated occasion level estimates of undifferentiated negative affect. **(A)** illustrates that aggregation, in general, leads to overestimation of undifferentiation, but it is minimized and constant using a Generalizability Theory approach compared to convention. **(B,C)** depict how individual item variance within subscale, and subscale variance within specific moments of different days, respectively, can bias undifferentiation estimates when unaccounted for, and this is again minimized using a GT approach. In all cases, ignoring the true momentary signal due to undifferentiation (solid black line) leads to bias, underscoring the need for momentary measures that disambiguate sources of variance.

To assess the degree to which these sources of variance would bias estimates of the true underlying undifferentiation value, we simulated data based on the observed variance component structure from the full GT model in Table 2. We then independently varied the amount of systematic variance due to day, scale^*^item, and scale^*^occasion^*^day from zero to approximately twice of what was empirically observed, because they were the largest sources of variance contained within each of the components of the conventional model. For each level of each manipulated variance component we: (1) generated 1,000 samples of 100 individuals, (2) performed variance decompositions according to the conventional, full GT, and occasion-level (which were then aggregated) approaches, and (3) calculated estimates of UA. Based on the simulation values we also calculated the true empirical undifferentiation estimate. Figure 2 shows the results of the simulations for each manipulated variance component with vertical lines indicating the amount of variance in the specific component that was empirically observed.

In general, when there is systematic variability due to day we see that the GT estimate is a slightly biased, but consistent estimator of the underlying ICC value.^7^ Similarly, the aggregated occasion-level estimate is slightly more biased than the GT estimate but also is consistent. In contrast, the conventional estimate is substantially more biased (25% more variance) when there is zero day-level variability, and that bias increases as the amount of day variability in the data increases.

When instead we vary the amount of variance due to particular items within a given subscale we see again that the GT estimate is a slightly biased but still consistent estimator of the underlying ICC estimate. In contrast, we see that the aggregated occasion-level estimate is the least biased of the three when there is no subscale-by-item variance, but as that variance increases it becomes increasingly biased.

Yet another pattern is observed when we vary the amount of variance associated with scale^*^occasion^*^day. Again, the GT estimate is on average the least biased and consistent across a range of possible values. As with day variability, as scale^*^occasion^*^day variability increases so does the already large bias in the conventional estimate. By comparison, the aggregated occasion-level estimate is most biased when there is no scale^*^occasion^*^day variability, but becomes less biased as such variability increases.

### Effects of undifferentiated affect on impulsivity and substance use

We next used the various UNA estimates from the example data as predictors in person-level and occasion-level regression models of impulsivity and substance use. Table 3 presents the results from the person-level analysis. UNA estimates were positively associated with reported impulsivity for each of the aggregated occasion level estimates. Importantly, the effect was weakest when occasions with zero item variance were imputed as undifferentiated (*β = 0*.16, *p* = 0.088), stronger when such occasions were left missing (*β = 0*.24, *p* = 0.004), and strongest when zero variance occasions were imputed as completely undifferentiated if negative affect was elevated and completely differentiated if negative affect was at floor levels (i.e., all 1’s; *β = 0*.30, *p* < 0.001). UNA was also positively associated with marijuana use using the conventional ICC approach (*β = 0*.21, *p* = 0.030) and imputing all zero variance occasions as complete undifferentiation (*β = 0*.25, *p* = 0.018). Using Erbas et al. (2022) approach, none of the associations were statistically significant, though the effect on marijuana use was consistent with the other indices but smaller.

**Table 3:** Person-level associations between undifferentiated negative affect and impulsivity/substance use using conventional and GT-based methods.

Variable	Person Level						Occasion level aggregated to person level											
	Conventional ICC			GT-based ICC			Erbas et al. (2022)			Impute as Undifferentiation			No Imputation			Conditional Imputation		
	*Est*	*SE*	*β*	*Est*	*SE*	*β*	*Est*	*SE*	*β*	*Est*	*SE*	*β*	*Est*	*SE*	*β*	*Est*	*SE*	*β*
Impulsivity	1.03	1.09	0.10	0.73	0.66	0.12	0.020	0.06	0.03	1.30^†^	0.76	0.16	2.09^**^	0.71	0.24	2.51^***^	0.60	0.30
Alcohol	0.05	0.05	0.10	0.00	0.03	0.00	0.002	0.004	0.07	0.00	0.05	0.00	0.00	0.05	0.00	0.00	0.04	0.02
Caffeine	−0.03	0.12	−0.03	0.03	0.08	0.04	0.001	0.001	0.00	0.00	0.12	0.00	−0.04	0.12	−0.03	−0.11	0.11	−0.09
Tobacco	−0.14	0.23	−0.06	−0.11	0.15	−0.07	0.010	0.019	0.06	0.08	0.22	0.04	−0.05	0.22	−0.02	−0.16	0.20	−0.07
Marijuana	0.17^*^	0.08	0.21	0.07	0.05	0.15	0.009	0.007	0.14	0.19^*^	0.08	0.25	0.07	0.08	0.09	−0.07	0.07	−0.08

^†^*p* < 0.10, ^*^p < 0.05, ^**^p < 0.01, and ^***^p < 0.001.

Importantly, as has been done in past research (Kashdan et al., 2010; Pond et al., 2012; Tomko et al., 2015), we explored whether UNA interacted with overall level of negative affect in predicting the various outcomes. In predicting impulsivity, the interaction was significant using the conventional (*b* = 4.37, *SE* = 0.98, *p* < 0.001, β = 0.33), GT-based (*b* = 2.55, *SE* = 0.71, *p* = 0.005, β = 0.28), complete undifferentiation (*b* = 4.17, *SE* = 1.11, *p* < 0.001, β = 0.29), and no imputation (*b* = 3.15, *SE* = 1.22, *p* = 0.011, β = 0.20) estimates, but not for the conditional imputation estimates (*b* = 1.44, *SE* = 1.19, *p* = 0.229, β = 0.09), which we note recodes occasions with all 1’s (“not at all”) for the negative affect items as completely differentiated. For all of the substances, all of the interaction effects were non-significant (all *p*’s > 0.100) except when using the conditionally imputed estimates to predict marijuana use (*b* = 0.43, *SE* = 0.13, *p* = 0.001, β = 0.29).

To further demonstrate the potential utility of our GT-based approach to calculating UA we also conducted momentary analyses, which included effects at the momentary, day, and person level in predicting momentary impulsivity and substance use—something that could not previously be done using conventional approaches. Table 4 shows the results of these analyses in which impulsivity was modeled continuously, whereas substance use was modeled dichotomously. UNA at all three levels of analysis (momentary, day, and person) was associated with momentary impulsivity, and this was generally robust to the different ways of handling the zero variance occasions. As in the person level analyses, the interaction between UNA and level of negative affect was significant, but only at the person level, and only for the undifferentiation imputed (*b* = 4.17, *SE* = 1.10, *p* < 0.001) and no imputation (*b* = 3.23, *SE* = 1.27, *p* = 0.013) methods. The exception was for the Erbas et al. (2022) method where only momentary undifferentiation was significant in the opposing direction (*b* = −0.006, *SE* = 0.003, *p* = 0.039), but the momentary interaction was significant (*b* = 0.008, *SE* = 0.002, *p* < 0.001) such that the overall effect becomes positive at approximately positive one half of a standard deviation of momentary variability.

**Table 4:** Occasion-level associations between undifferentiated negative affect and impulsivity/substance use using occasion level ICC.

Variable	Level	Impute as Undifferentiation		No imputation		Conditional imputation		Erbas et al. (2022)	
		*Est*	*95% CI*	*Est*	*95% CI*	*Est*	*95% CI*	*Est*	*95% CI*
Impulsivity	Occasion	0.20^**^	[0.07, 0.33]	0.22^**^	[0.08, 0.37]	0.27^***^	[0.16, 0.37]	−0.006^*^	[−0.011,-0.000]
	Day	0.42^***^	[0.20, 0.65]	0.70^***^	[0.42, 0.98]	0.61^***^	[−0.34, 0.87]	−0.004	[−0.014,0.007]
	Person	1.31^†^	[−0.19, 2.82]	2.27^**^	[0.79, 3.75]	2.51^***^	[1.31, 3.72]	0.018	[−0.095,0.131]
Alcohol^a^	Occasion	1.24	[0.92, 1.68]	1.12	[0.82, 1.52]	1.01	[0.81, 1.25]	0.997	[0.991,1.004]
	Day	1.42^†^	[0.94, 2.12]	1.47^†^	[0.99, 2.20]	1.35	[0.91, 2.01]	1.003	[0.989,1.017]
	Person	1.06	[0.15, 7.60]	1.32	[0.20, 8.85]	1.43	[0.33, 6.13]	1.038	[0.923,1.168]
Caffeine^a^	Occasion	0.82^**^	[0.72, 0.93]	0.86^*^	[0.74, 0.99]	1.08	[0.97, 1.20]	0.995^*^	[0.990,0.999]
	Day	0.86	[0.72, 1.03]	0.96	[0.80, 1.15]	1.05	[0.91, 1.22]	0.996	[0.988,1.004]
	Person	1.01	[0.34, 2.94]	0.84	[0.30, 2.33]	0.55	[0.21, 1.42]	1.009	[0.924,1.102]
Tobacco^a^	Occasion	0.95	[0.88, 1.03]	0.98	[0.90, 1.07]	1.02	[0.96, 1.09]	0.999	[0.995,1.002]
	Day	0.84^**^	[0.74, 0.96]	0.86^*^	[0.75, 0.98]	1.00	[0.89, 1.12]	1.002	[0.989,1.014]
	Person	1.56	[0.22, 10.81]	1.18	[0.16, 8.52]	0.54	[0.09, 3.20]	1.005	[0.861,1.174]
Marijuana^a^	Occasion	0.80	[0.60, 1.07]	0.91	[0.68, 1.22]	1.15	[0.95, 1.39]	0.995	[0.986,1.003]
	Day	0.77	[0.46, 1.27]	0.90	[0.54, 1.52]	1.13	[0.72, 1.75]	1.002	[0.981,1.024]
	Person	390.92^***^	[13.59, 11244.11]	26.87^†^	[0.73, 989.01]	0.35	[0.01, 11.27]	1.195	[0.929,1.538]

^†^*p* < 0.10, ^*^p < 0.05, ^**^p < 0.01, ^***^p < 0.001. CI, confidence interval. ^a^Parameter estimates and confidence intervals are odds ratios.

Although in the person-level analyses we did not observe associations between UNA and either alcohol, caffeine, or tobacco use, when we analyzed the data at a more granular level we did observe a number of significant effects. There was a trend such that on days when individuals felt more undifferentiated than average they were more likely to drink alcohol for both the undifferentiation imputed (*OR* = 1.42, 95% *CI* = [0.94, 2.12], *p* = 0.093) and no imputation (*OR* = 1.47, 95% *CI* = [0.99, 2.20], *p* = 0.059) operationalizations. This was not the case for the conditional imputation method (*OR* = 1.35, 95% *CI* = [0.91, 2.01], *p* = 0.140), though the effect was in the same direction, suggesting that the recoding did not have a strong impact on this effect. This was corroborated by the absence of an interaction effect between UNA and overall level of negative affect at the day level (*OR* = 1.07, 95% *CI* = [0.33, 3.45], *p* = 0.915).

Also at the day level, when individuals were feeling more undifferentiated on a given day they were less likely to smoke tobacco for the undifferentiation imputed (*OR* = 0.84, 95% *CI* = [0.74, 0.96], *p* = 0.008) and no imputation (*OR* = 0.86, 95% *CI* = [0.75, 0.98], *p* = 0.025) operationalizations, but not for the conditional imputation method (*OR* = 1.00, 95% *CI* = [0.89, 1.12], *p* = 0.978). Again, we did not observe an interaction effect between undifferentiated negative affect and level of negative affect for this effect when using the conditional imputation (*OR* = 0.94, 95% *CI* = [0.21, 4.14], *p* = 0.933).

In a similar direction as tobacco but at the momentary level, we did observe a significant effect for increased momentary feelings of UNA being associated with less caffeine use, again for the undifferentiation imputed (*OR* = 0.82, 95% *CI* = [0.74, 0.93], *p* = 0.003), no imputation (*OR* = 0.86, 95% *CI* = [0.74, 0.99], *p* = 0.035), and (Erbas et al., 2022 *OR* = 0.995, 95% *CI* = [0.990, 0.999], *p* = 0.019) methods. Interestingly, the same effect for the conditional imputation began to trend in the *opposite* direction (*OR* = 1.08, 95% *CI* = [0.97, 1.20], *p* = 0.155), as did the corresponding interaction between UNA and level of negative affect (*OR* = 0.77, 95% *CI* = [0.53, 1.12], *p* = 0.167), which, consistent with the other two imputation methods, would suggest a decrease in caffeine use at higher levels of UNA and higher overall negative affect.

Replicating the person level analyses, we observed a significant effect such that people who, across the EMA period, were more undifferentiated were also more likely to smoke marijuana at any given occasion for both the undifferentiation imputed (*OR* = 390.92, 95% *CI* = [13.59, 11244.11], *p* < 0.001) and no imputation (*OR* = 26.87, 95% *CI* = [0.73, 989.01], *p* = 0.074) methods, but not for the conditional imputation (*OR* = 0.35, 95% *CI* = [0.01, 11.27], *p* = 0.557) variation. This last effect, however, as in the person level analyses, was qualified by a significant interaction with level of negative affect (*OR* = 5727.303, 95% *CI* = [41.17, 796912.37], *p* < 0.001) while the other two were not (*p*’s > 0.325).

**Figure 3:**
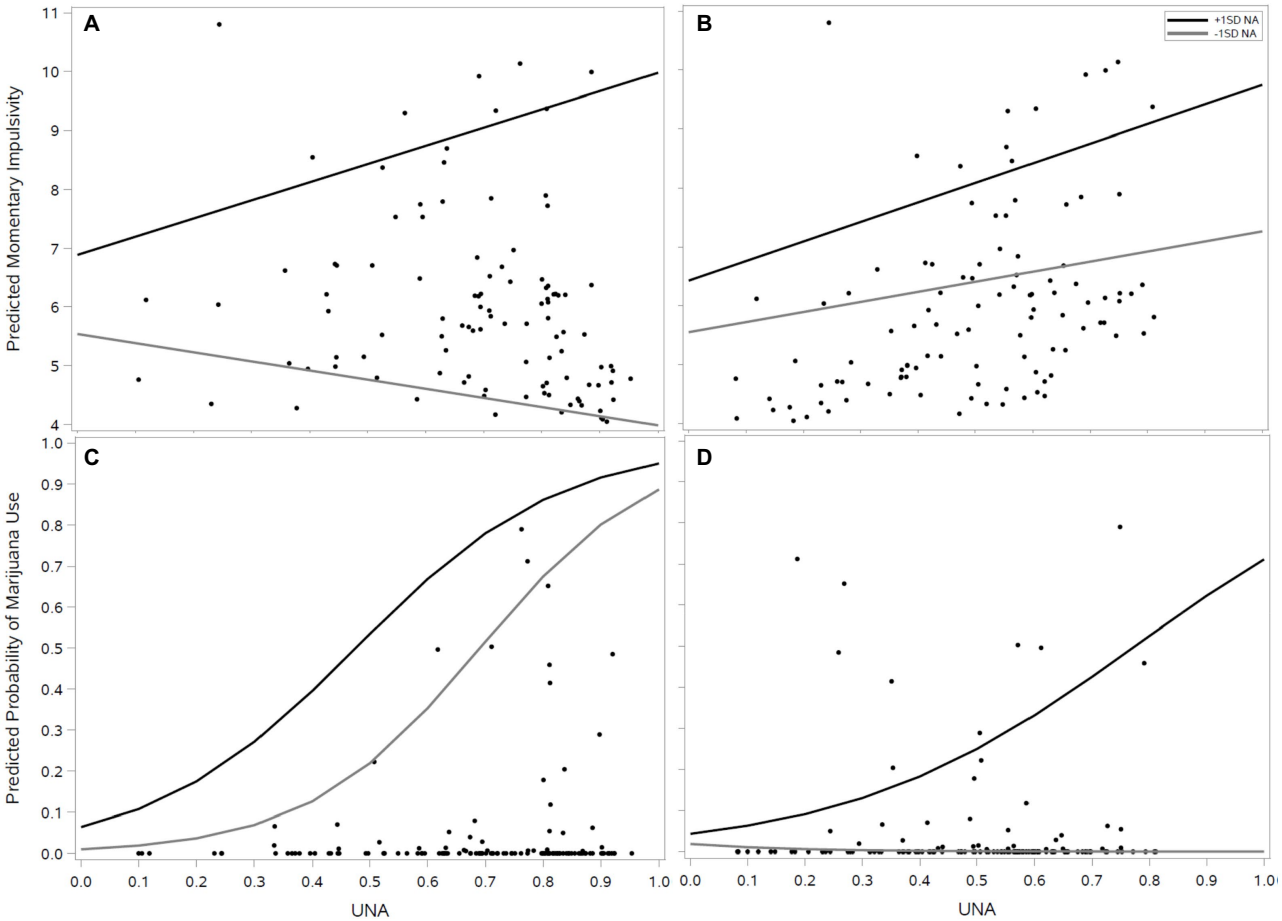
Interaction plots (undifferentiated negative affect by level of negative affect) for impulsivity **(A,B)** and marijuana use **(C,D)** using either undifferentiated imputation **(A,C)** or conditional imputation **(B,D)**. **(A,B)** depict the interaction **(A)** versus main **(C)** effect of negative affect in tandem with undifferentiated negative affect when treating zero variance floor responses as complete undifferentiation **(A)** versus differentiation **(C)**, respectively. **(C,D)** show the same comparison but with respect to marijuana use, and how an interaction that is not otherwise observed in the traditional treatment (equivocating zero variance with undifferentiation); **(C)** is revealed when treating zero variance floor ratings as being differentiated **(D)**.

Figure 3 illustrates the interaction effects at the person level for momentary impulsivity (Panels A and B) and marijuana use (Panels C and D) when using undifferentiated imputation (Panels A and C) versus conditional imputation (Panels B and D), because the two methods represent contrasting ways of coding zero variance occasions. The analogous plots of the interaction effects for the aggregated between-person analyses looked very similar. Individual data points represent raw person-level aggregates to minimize saturation while illustrating the impact of the different methods for handling zero variance occasions. For impulsivity, zero variance occasions coded as completely undifferentiated lead to a cluster of points in the bottom right of Panel A, which have high average UNA and drive the interaction effect. In contrast, when those occasions that have zero variability and are at the floor of the scale are instead coded as complete differentiation (with elevated zero variance occasions being coded as complete undifferentiation) the distribution of points become more evenly spread and we observe two positive main effects for UNA and level of negative affect.

For marijuana use, we see that the positive effect of undifferentiated negative affect is largely driven by a group of 10 individuals who used marijuana daily and tended to have high UNA (Panel C). However, these individuals were more than twice as likely to have zero variance occasions rated at the floor of the scale (*OR* = 2.42, 95% *CI* = [1.02, 5.75], *p* = 0.045), which drove the main effect. When those occasions are instead coded as completely differentiated that group of individuals is more evenly distributed across the UNA range, and we can more clearly see that the impact of UNA is conditional on NA levels being higher.^8^

## Discussion

In the current empirical example and simulations we sought to, (1) refine the estimation methods previously used to characterize UA, (2) extend those methods beyond the person-level to more granular levels of experience where UA is theorized to operate, and (3) consider how certain consequences of the statistical estimation of UA may have more nuanced implications for its theoretical interpretation. We next discuss our findings with respect to each of these goals and make suggestions for future research on UA.

### Refining the estimation of undifferentiated affect

In general, we observed that conventional estimates of UA may be inflated as a result of confounding other systematic sources of variance with that observed at the item level. We would argue that these sources of variance should not be included in what is considered signal for UA because they may be more parsimoniously explained by other emotion regulation processes such as circadian rhythms (e.g., Larsen, 1985; Rusting and Larsen, 1998) or by individual differences in reporting bias (Podsakoff et al., 2003). Furthermore, such external sources of variance may distort observed associations between UA and other variables. Our procedure for calculating UA at the person level did produce estimates that correlate highly with conventional approaches; however, based on the presented simulations this does not have to be the case, and our approach would be preferred because it is both more precise and robust. At the momentary level, our procedure correlated less highly with conventional approaches, both because of differences in the level of estimation and limiting factors implicit in estimating UA at the momentary level (i.e., empirically cannot estimate day level variation). We suggest that researchers use the approach that corresponds to their desired level of analysis (i.e., person level GT for person level hypotheses, occasion level GT for more dynamic or contextualized occasion level hypotheses). For occasion level applications, adjusting for known influences like time of the day and day of the week will mitigate variance confounded within the estimate of the error for individual UA values.

### Extending UA measurement to the momentary level

Past studies have attempted to estimate UA at the level of the person, often aggregating across multiple daily reports and/or reports within a given day (e.g., Demiralp et al., 2012; Erbas et al., 2018; Kalokerinos et al., 2019). Recently, methods have been proposed to estimate differentiation at the momentary level, though still focusing on individual emotion items (Erbas et al., 2022). However, in light of the fact that many affect scales contain multiple items that correspond to individual subscales, we were able to leverage between-and within-subscale variation to estimate UA at the momentary level whereas other momentary indices explicitly confound them. To that end, the proposed approach can be used in other contexts outside of EMA, including experimental studies, panel studies, and even one-shot observational/correlational studies. Because the method can be used to estimate undifferentiated affect within a given moment, what constitutes a “moment” can be very general with respect to timing. Importantly, this requires multiple items to be assessed for each subscale so that consistency in ratings across subscales can be compared to that which is observed within them for a given reporting occasion. In our example, we assessed between five and six items for the hostility, sadness, and fear subscales within overall negative affect. In contrast, the 10 items also collected in the protocol corresponding to positive affect were not *a priori* chosen to assess multiple positive affect subscales. As a result, we would not be able to estimate undifferentiated positive affect at the momentary level without grouping those items into at least two subscales. We note however, that we would be able to estimate *daily* undifferentiated positive affect given that those items were each assessed approximately six times per day, allowing us to cross item and occasion in decomposing the variance.

We believe estimating UA at the momentary (and also daily) level confers distinct advantages because that is where the process of reflecting on one’s emotional state and choosing appropriate regulation strategies is theorized to operate (Barrett et al., 2001). This allows for the moment-to-moment dynamics of emotion differentiation as a process to be investigated and modeled, and for situational correlates of its experience to be explored. In our example, we demonstrated that only impulsivity and marijuana use correlated with UNA at the person level. However, moving to the momentary level, we observed at least trends between UNA and alcohol, caffeine, and tobacco use as well. That is, while being a high UNA person on average was not associated with substance use other than marijuana, we did observe that if people were higher in UNA on a particular day compared to their usual daily UNA they were somewhat more likely to drink alcohol and less likely to smoke tobacco. Furthermore, within a given day, if someone was experiencing more UNA compared to their UNA across the rest of the day he/she was less likely to consume caffeine. These are potentially interesting effects that give insight into the situation-specific versus diffuse aspects of UA itself, and how UA may be composed of a set of regulatory processes (e.g., Tomko et al., 2015). Critically, conventional person-level approaches would miss these more fine-grained associations, unless researchers had specific person-level hypotheses, in which case the conventional and GT approaches produce similar results in our empirical analyses while our simulations show that conventional ICC effect sizes will be attenuated. Though preliminary, these results suggest countervailing associations between UNA and alcohol/marijuana compared to UNA and caffeine/tobacco. We can speculate that this may relate to the initial sedative but combined sedative/stimulant effects of alcohol (Chung and Martin, 2009) and marijuana (Block et al., 1998) as a coping response to experiencing UNA, while the primarily stimulant effects of nicotine (Corrigall et al., 1992) and caffeine (Biaggioni et al., 1991) may be motivationally inhibiting when experiencing UNA. Other researchers have reported positive associations between alcohol use emotion differentiation (Kashdan et al., 2010), which lends credence to part of this interpretation, but further research and replication are necessary.

### Interpreting estimates of undifferentiated affect in the absence of elevated affect

In calculating UA at the momentary level, we encountered an estimation issue not encountered by other UA researchers who in the past have created estimates only at the person level. Namely, there were a large proportion of occasions (18.8%) with no variability in ratings, and so no estimate could be empirically derived. The absence of variability at the person level, over the course of multiple days or weeks, is unlikely, and so would rarely be expected to present a problem for person level estimation. However, the likelihood of sampling a person randomly during the day and he/she reporting that there is nothing negatively emotion-inducing going on (18.3% in our data) seems quite possible. These empirically “difficult” observations may also have theoretical ramifications for the conceptualization of UA. From a person level approach, these observations are implicitly counted as completely undifferentiated because they do not contribute any variance to systematic between-subscale differences, which will magnify estimates of UA (see Table 1). However, conceptually it is unclear if such instances correspond to the lack of emotional clarity/granularity (Suvak et al., 2011; Grühn et al., 2013) or generalized feelings of negativity (Barrett et al., 2001) on which UA is defined. We suggest that reporting floor responses of “Not at all” to all negative affect items may in fact indicate a *highly differentiated* absence of negativity.^9^ This alternate view, when recoded in the data, can greatly change the apparent pattern of results. The observation that this occurs so frequently in a sample of individuals with disorders characterized by chronic elevated negativity (American Psychiatric Association, 2013), suggests that it is likely more prevalent in healthy samples and could therefore be even more impactful. Moreover, as Figure 1 suggests, such floor responses across both all positive and negative items (4.1%) might constitute a separate affective state that might require additional considerations of imputation or missingness.

Past research has dutifully noted and replicated that there is often an interaction effect between UA and level of negative affect (Barrett et al., 2001; Kashdan et al., 2010; Pond et al., 2012; Selby et al., 2013; Zaki et al., 2013). This interaction effect in each of the reported studies is predominantly driven by an association between UA and the outcome of interest when affect intensity is elevated, with little to no (and sometimes reversed) association observed at low levels of affect. We suggest that the absence (or reversal) of an association at low levels of affective intensity may be because high UA is simply identifying a lack of variance at the floor of the affect scale (i.e., a participant consistently reporting that he/she is not feeling any affect). This could also explain possible reversals of identified associations in that highly “undifferentiated” scores at low affect levels may actually be more precise while lower scores at low affect levels may be the result of random slight elevations in a single affect item of a scale (i.e., it represents the only variance available, gets relegated to error, and results in a high estimate of UA).^10^

This is precisely the result observed in our example with impulsivity (Figures 3A,B). Panel A, which codes zero variance occasions as complete UA, displays the same interaction pattern observed in previous investigations.^11^ However, when zero variance occasions at the floor of the scale are coded as completely differentiated the result is two main effects. As mentioned earlier, the apparent reversal of this pattern for marijuana use is largely due to the tendency for daily marijuana users to increasingly report “Not at all” for all negative affect items on a given occasion, an effect that was otherwise attributed to all individuals when coding such observations as complete UA.

In sum, we do not contest previously reported interaction effects between UA and affect intensity, but suggest that rather than an interesting theoretical interaction, it may simply be driven by an absence of variance. Regardless, if investigators are using person level estimates of UA in analyses, or otherwise coding zero variance reports as complete undifferentiation, it is essential to also include the main effect of level of affect and their interaction. The alternative approach of coding zero variance occasions as complete differentiation may simplify interpretations, but the interaction should still be tested. We have no specific preference concerning which approach to use. Our goal is to elucidate an empirical wrinkle in the estimation of UA that may lead to alternative interpretations of some UA findings.

## Limitations

Despite the potential advantages of the suggested approach with respect to measuring affective differentiation more reliably and more in line with moment to moment experiential dynamics than previous operationalizations, there are inherent limitations with respect to conceptualization and generalizability. Undifferentiated emotion pertains to individual, specific emotion labels and individuals’ ability or tendency to disambiguate them when they experience an affectively arousing and valenced stimulus (Barrett et al., 2001). The current measure pertains to undifferentiated affect, which is broader in that it includes sets of neighboring individual emotions (Russell, 1980) and is inherently less well-defined theoretically. As a result, based on the construction of the PANAS-X, our conceptualization of undifferentiated affect is constrained to broader distinctions between hostility, fear, and sadness, and importantly also excludes other important categories of negative emotions such as guilt or jealousy. Our method cannot reliably disentangle specific differentiation across more individualized, nuanced emotions that are measured with single items, which we know to be inherently less reliable (Spearman, 1904, 1910). However, one option, if researchers collected data with the appropriate structure as illustrated using the GT framework, is to decompose the variance of ratings in this case at the day level since each item will then be assessed multiple times. The inherent limitation of this approach would be that undifferentiation is then necessarily estimated as a day level construct as opposed to a momentary one and lower level dynamics may be obscured. This could still have empirical utility to the extent that individuals do experience undifferentiation at the day level across contexts. Alternatively, researchers could expand or develop new measures that attempt to do what the PANAS-X does in terms of achieving relative consistency in ratings within subscales but with respect to more specific emotions.

In the current example context we are also limited in our possible generalization of the UA estimation method in terms of the affect measure (17 items) and the momentary assessment design (6 prompts per day over 28 days). Other common scales (e.g., POMS, McNair et al., 1992; MDMQ, Wilhelm and Schoebi, 2007) and designs include many fewer items and assessments (e.g., fixed, sparser, event-contingent), respectively, which may have systematic, unappreciated effects on UA estimation as a function of diurnal or weekly cycles when calculated at the person versus momentary level. These factors are also expected to be related to considerations regarding participant burden when deciding on measures and sampling schemes beyond appropriately capturing affective processes (Eisele et al., 2021). Moreover, if the specific affect differentiation of interest is broader (e.g., positive versus negative) or with respect to specific emotion categories (e.g., hostility, anxiety) fewer items may be necessary.

## Conclusion

The construct of emotion differentiation is receiving more attention as a core component of emotion regulation in both healthy and clinical individuals (Kashdan et al., 2015). It is viewed as a critical gateway toward the identification and mobilization of emotional coping and eventual mental health. The precise measurement of emotion differentiation and understanding the connection between its theoretical conceptualization and empirical operationalization is essential for characterizing *how* emotion differentiation facilitates such processes in everyday life. We suggest refinements in how emotion differentiation is estimated and present a new method for estimating it at the level of individual experience. These advancements allow researchers to rule out correlated regulatory processes, make fuller use of available data, and map out emotion differentiation as its own dynamic process that may change across different environmental contexts.

## Data availability statement

The data analyzed in this study are subject to the following licenses/restrictions: Data are still held under the aims of the granting agency. Requests to access these datasets should be directed to trullt@missouri.edu.

## Ethics statement

The studies involving human participants were reviewed and approved by University of Missouri Institutional Review Board. The patients/participants provided their written informed consent to participate in this study.

## Author contributions

SL designed and performed the research and analyzed the data. SL and TT interpreted the results and wrote the paper. All authors contributed to the article and approved the submitted version.

## Funding

This research was supported by the National Institutes of Health grants R21 MH069472 (TT), P60 AA1198 (Heath), T32 AA013526 (Sher), and R01 AA027264 (SL and Hennes).

## Conflict of interest

The authors declare that the research was conducted in the absence of any commercial or financial relationships that could be construed as a potential conflict of interest.

## Publisher’s note

All claims expressed in this article are solely those of the authors and do not necessarily represent those of their affiliated organizations, or those of the publisher, the editors and the reviewers. Any product that may be evaluated in this article, or claim that may be made by its manufacturer, is not guaranteed or endorsed by the publisher.

## Footnotes

^1^We note that there can be some ambiguity as to which terms should be included in the model. For example, the items for each subscale are likely unique to that subscale and so, formally, item is nested within subscale. In this case the terms *(IS)_is_*, *(ISO)_is_*, and *(ISD)_is_* would be removed from the model and*I*would be replaced with *I_i(s)_*. However, to the extent to which the items are consistently given in the same order when presented, there may be a systematic ordering effect (e.g., the first item is disproportionately elevated; Knowles et al., 1996) that allows the interaction effect to be estimated. Similarly, conventional parlance would suggest that occasions are nested within days; however, if individuals are systematically sampled in the morning, afternoon, and evening across multiple days, occasions can be considered crossed with days (i.e., the interaction can be estimated). Some of the terms may be inestimable, such as the higher order interactions, in which case they may often be dropped with little effect. If a researcher’s design does not conform to this model they may simply drop all terms containing that design element (i.e., occasions) and perform a variance decomposition as before.

^2^If the number of items varies by subscale, a conservative estimate would be to use the smallest item set size. Alternatively, a harmonic mean could be estimated (Raudenbush and Bryk, 2002).

^3^Results from the current sample with different foci are also published in Jahng et al., 2008, 2011; Trull et al., 2008; Solhan et al., 2009; Tomko et al., 2014,^2015^.

^4^Of all caffeine use reports 997 (17.2%) had no variability in negative affect item reports, 1,271 (18.4%) had no variability for tobacco use reports, 179 (18.9%) had no variability for alcohol use reports, and 200 (24.4%) had no variability for marijuana use reports.

^5^In addition to the primary variables of interest listed in the model equation, we adjusted for other factors that might be systematically related to the dependent variables. For momentary analyses, this included day of the week and time of the day. In both momentary and person level analyses, we also included reports of conflict, being rejected, being complimented, or health problems.

^6^This includes the 847 occasions where there was also no variability in positive affect ratings. If we instead impute those 847 occasions as complete undifferentiation or exclude them from the analyses (i.e., missing) the pattern of results does not change.

^7^This bias is due to positive skew in the underlying distributions of each of the variance components, which are bounded below by 0. Increasing the relative size of each of the variance components gradually mitigates this, but we elected to retain more realistic values so that the results may be more meaningfully compared.

^8^The group of 10 individuals who were daily marijuana users also had a marginal tendency to report elevated negative affect (*b* = 0.52, *SE* = 0.29, *p* = 0.076).

^9^This interpretation is supported and suggests that these reports are valid given the vast majority of them that are paired with positive affect reports that have variability (Figure 1).

^10^This scenario composed 21.0% (*n* = 4,064) of all occasions in our example data set, in addition to the 18.3% of rating sets as all 1’s.

^11^Note that the person level analysis (see Table 3 and results section), which does not explicitly code zero variance occasions as UA but rather does so implicitly, produces nearly identical results. This effect is not due to the level of analysis.
